# The Orphan Gene *ybjN* Conveys Pleiotropic Effects on Multicellular Behavior and Survival of *Escherichia coli*

**DOI:** 10.1371/journal.pone.0025293

**Published:** 2011-09-27

**Authors:** Dongping Wang, Bernarda Calla, Sornkanok Vimolmangkang, Xia Wu, Schuyler S. Korban, Steven C. Huber, Steven J. Clough, Youfu Zhao

**Affiliations:** 1 Department of Crop Sciences, University of Illinois, Urbana, Illinois, United States of America; 2 Department of Natural Resources and Environmental Sciences, University of Illinois, Urbana, Illinois, United States of America; 3 Program in Physiological and Molecular Plant Biology, University of Illinois, Urbana, Illinois, United States of America; 4 Agricultural Research Service, United States Department of Agriculture (USDA), Urbana, Illinois, United States of America; University of Wisconsin-Milwaukee, United States of America

## Abstract

*YbjN*, encoding an enterobacteria-specific protein, is a multicopy suppressor of temperature sensitivity in the ts9 mutant strain of *Escherichia coli*. In this study, we further explored the role(s) of *ybjN*. First, we demonstrated that the *ybjN* transcript was about 10-fold lower in the ts9 strain compared to that of *E. coli* strain BW25113 (BW). Introduction of multiple copies of *ybjN* in the ts9 strain resulted in over-expression of *ybjN* by about 10-fold as compared to that of BW. These results suggested that temperature sensitivity of the ts9 mutant of *E. coli* may be related to expression levels of *ybjN*. Characterization of *E. coli ybjN* mutant revealed that *ybjN* mutation resulted in pleiotropic phenotypes, including increased motility, fimbriation (auto-aggregation), exopolysaccharide production, and biofilm formation. In contrast, over-expression of *ybjN* (in terms of multiple copies) resulted in reduced motility, fimbriation, exopolysaccharide production, biofilm formation and acid resistance. In addition, our results indicate that a *ybjN*-homolog gene from *Erwinia amylovora*, a plant enterobacterial pathogen, is functionally conserved with that of *E. coli*, suggesting similar evolution of the YbjN family proteins in enterobacteria. A microarray study revealed that the expression level of *ybjN* was inversely correlated with the expression of flagellar, fimbrial and acid resistance genes. Over-expression of *ybjN* significantly down-regulated genes involved in citric acid cycle, glycolysis, the glyoxylate shunt, oxidative phosphorylation, amino acid and nucleotide metabolism. Furthermore, over-expression of *ybjN* up-regulated toxin-antitoxin modules, the SOS response pathway, cold shock and starvation induced transporter genes. Collectively, these results suggest that YbjN may play important roles in regulating bacterial multicellular behavior, metabolism, and survival under stress conditions in *E. coli*. These results also suggest that *ybjN* over-expression-related temperature rescue of the ts9 mutant may be due to down-regulation of metabolic activity and activation of stress response genes in the ts9 mutant.

## Introduction

Microorganisms survive in different environmental conditions by adaptation via diverse metabolic pathways. Microbial genome projects have indicated that the function of about half of the genes of any given bacterial genome is still unknown [1], [2]. These are annotated as genes of unknown function, and their products are typically referred to as ‘conserved’ or ‘hypothetical’ proteins. As one of the best-studied prokaryotic model organisms, *Escherichia coli* strain K12 still has around 2000 out of 4377 genes which have not been characterized [3]. Characterization of these unknown proteins remains a major challenge and only around 30 new *E. coli* genes have been experimentally characterized each year [4]. At the current pace, it would take several decades before the biological functions of all the uncharacterized genes are determined. On the other hand, these unknown proteins also provide opportunities for us to better understanding the biology of a particular organism, and open up potentially new biomedical and commercial opportunities [5].

Orphan genes (ORFans) are annotated genes that exist exclusively within a particular genome, strain, species, or lineage. Often, orphan genes are of similar size (400–600 bp), and they are significantly shorter than genes (800–1000 bp) with heterogeneous occurrence in distantly-related prokaryotic species [6]. To study the function of ORFans, a comparative genomic approach is often not feasible. One such ORFan is the enterobacteria-specific gene *ybjN* [7]. However, our knowledge of the function of the *ybjN* gene and/or its functional conservation among enteric bacteria is limited.

Chen *et al.* (2006) have reported that over-expression (multiple copies) of *ybjN* suppresses temperature sensitivity conferred by point mutations in the *coaA* gene in *E. coli* ts9 strain, which can only grow at 30°C [8]. In addition, temperature-sensitivity caused by other point mutations, such as those of *coaA14* (DV51 strain), *coaA15* (DV70 strain) and *ilu*-1 can also be rescued by *ybjN* over-expression. However, these rescued strains can only grow at 37°C, but not at 40°C, the temperature at which most ts9 spontaneous revertants can grow, indicating that the rescued strain is not the result of reversion of the point mutation. These observations have led the authors to propose that YbjN may function as a general stabilizer for some unstable proteins. However, no interacting proteins for YbjN were found in a recent pull-down assay of *E. coli* K12 strain [9]. It was reported that expression of *ybjN* is up-regulated by several fold when *marA*, a transcriptional activator of antibiotic resistance, is constitutively expressed [10]. Microarray analysis revealed that *ybjN* expresses at a high level in *E. coli* under various stress conditions [11], [12], [13]. These results suggest that YbjN may be a general stress response gene or a “survival” gene.

Recent studies have further indicated that *ybjN* may play a role in bacteria-host interactions and virulence. Whole-genome expression profiling has revealed that *ybjN* is significantly induced in *E. coli* during growth on mucus, conditions designed to mimic the human intestine [14]. Following human macrophage infection, expression of the *ybjN* increased by 3-fold in enterohemorrhagic *E. coli* O157:H7 [15]. Previously, we have reported that an YbjN homolog in *Erwinia amylovora*, a plant enterobacterial pathogen causing fire blight of apples and pears, negatively regulates amylovoran production, which is a major virulence factor [16]. Mutations in the *ybjN* resulted in slightly increased virulence as compared to that of the wild type strain (Zhao, unpublished data). These results strongly suggest that YbjN may be required for regulation of bacterial virulence factors and for establishment and/or maintenance of bacteria–host interaction.

The objectives of this study include the following: characterize the effects of *ybjN* knockout in *E. coli*; further determine how over-expression of *ybjN* affects *E. coli* under various stress conditions as well as its multicellular behaviors; and determine how *ybjN* knockout and over-expression influence global gene expression in *E. coli*.

## Results

### The orphan gene ybjN is a multi-copy suppressor of coaA-associated temperature sensitivity in the ts9 mutant of E. coli

**Figure 1:**
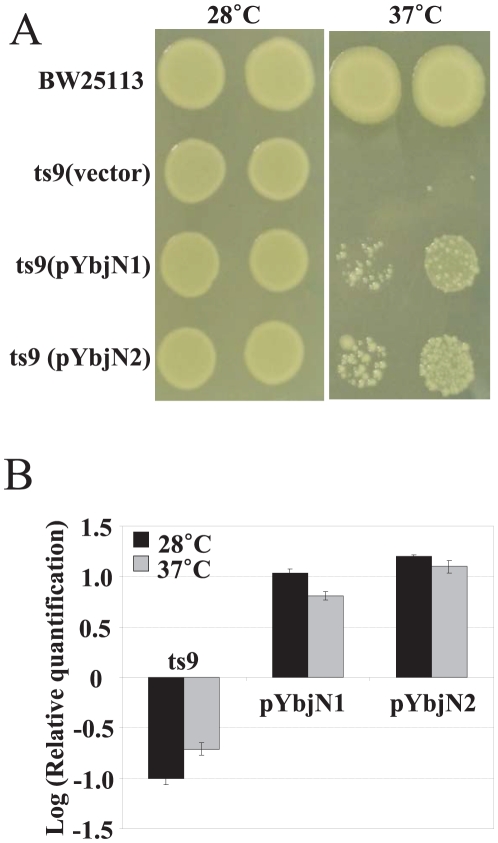
Expression level of *ybjN* is directly related to its ability to rescue the temperature sensitivity of *Escherichia coli* ts9 mutant. A. Over-expression of *ybjN* from both *E. coli* and *Erwinia amylovora* rescued the temperature sensitivity of ts9 mutant of *E. coli*. BW: *E. coli* strain BW25113; pYbjN1 and pYbjN2: *E. coli* and *E. amylovora ybjN* gene in *pGEM T easy* vector, respectively; Empty vector (*pGEM T easy*) as negative control. Columns represented two dilutions of inoculum at both temperatures. B. Relative expression of *ybjN* gene in the ts9 mutant and the ts9 mutant strain harboring high copy plasmids of *ybjN* gene from both *E. coli* and *E. amylovora* compared to the *E. coli* strain BW25113 by qRT-PCR, normalized to the expression value of 16S rRNA (*rrsA*) gene. Cells were grown in LB broth with shaking overnight at 28°C and 6 h at 37°C. ts9: *E. coli* ts9 mutant; pYbjN1: ts9 over-expressing *E. coli ybjN* gene; pYbjN2: ts9 overexpressing *E. amylovora ybjN* gene.

In a previous genetic screening, it has been reported that *E. coli ybjN* gene is a multi-copy suppressor of the *coaA*-associated temperature sensitivity in the ts9 mutant [8]. To reconfirm this result and to determine whether a homolog of *ybjN* from *E. amylovora* has a similar function to that of *E. coli*, multicopy plasmids containing *ybjN* from both *E. coli* and *E. amylovora* were introduced into the ts9 mutant of *E. coli*. The ts9 mutant strain with an empty vector can only grow at 28°C, but not at 37°C; while the *E. coli* strain BW25113 (BW) can grow at both temperatures (Fig. 1A). Similar to previous findings, the ts9 strain harboring high copy numbers of both *E. coli* and *E. amylovora ybjN* genes grew at 37°C (Fig. 1A), but not at 40°C (data not shown). These findings have re-confirmed previous reports that the *ybjN* is a multi-copy suppressor of temperature sensitivity in the ts9 strain [8]. These results have also indicated that *E. amylovora ybjN* functions similarly as that of *E. coli*.

To determine whether the rescue of temperature sensitivity in *E. coli* ts9 mutant is due to over-expression of *ybjN* transcripts, qRT-PCR was conducted to quantify the relative expression of *ybjN* gene in BW, ts9 strain, and ts9 strain harboring multiple copies of the *ybjN* gene (Fig. 1B). The *ybjN* transcript was around 10-fold lower in ts9 strain compared to that of the BW strain. When multiple copies of the *ybjN* gene were introduced into the ts9 strain, expression levels of *ybjN* were 10- and 100-folds higher than those of the BW and the ts9 strain, respectively. These results indicate that the *ybjN* gene is significantly down-regulated in the ts9 strain and multiple copies of the *ybjN* genes lead to over-expression of *ybjN*. This also suggests that the temperature sensitivity of the *E. coli* ts9 mutant may be related to expression levels of *ybjN*.

### Homologues of ybjN gene are detected only in Enterobacteriaceae

Both *E. coli* and *E. amylovora ybjN* genes are very small, encoding highly conserved proteins of 158 and 161 amino acids, respectively. A Basic Local Alignment Search Tool (blastp) has been used to determine distribution of YbjN [17]. As listed in Table S1, YbjN orthologs are conserved in Enterobacteriaceae. The gene context surrounding the *ybjN* gene has also been determined in various enterobacteria. Downstream of the *ybjN* orthologues is the *potFGHI* operon, which is predicted to encode a putrescine ABC transport system (Fig. S1). In both *E. amylovora* and *Erwinia tasmaniensis*, upstream of *ybjN* loci is the *nfsA* gene, which encodes a nitroreductase. Whereas, the *rimK* gene, involved in ribosomal S6 protein modification, is located between *ybjN* and *nfsA* in *E. coli*, *Salmonella enterica*, *Pectobacterium carotovorum* and *Dickeya dadantii*. Unlike *E. coli*, *Yersinia pestis* lacks both *nfsA* and *rimK* genes, and replaces them with a gene encoding a putative membrane protein. Deduced amino acid sequence alignments are shown in Fig. S2.

### Over-expression of ybjN suppresses bacterial growth in liquid media

**Figure 2:**
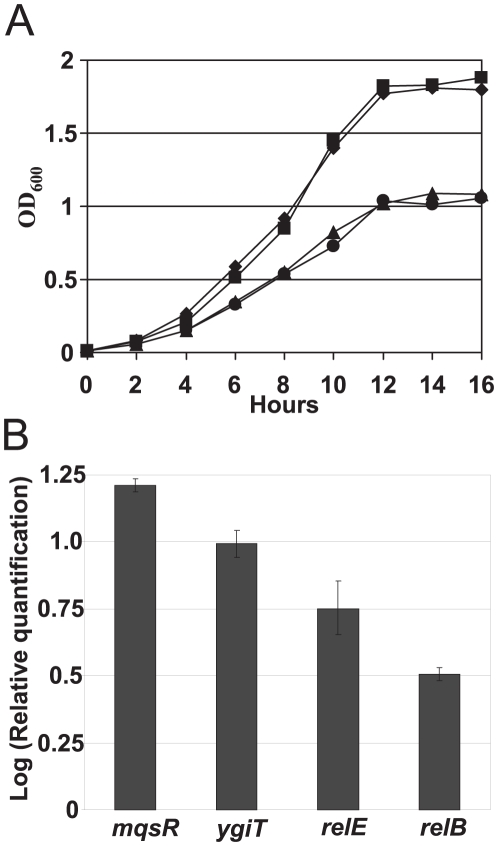
Over-expression of *ybjN* leads to growth inhibition and up-regulation of toxin-antitoxin modules. A. Growth curve of *ybjN* mutant and strain over-expressing *ybjN* in LB broth at 37°C. ♦: *E. coli* strain BW25113 (BW), ▪: Δ*ybjN mutant*, ▴: *E. coli* strain BW25113 (pYbjN1), •: *E. coli* strain BW25113 (pYbjN2). pYbjN1 and pYbjN2: *E. coli* and *E. amylovora ybjN* gene in *pGEM T easy* vector, respectively; original inoculum at an OD_600_ of 0.001. B. Relative expression of *mqsR*, *ygiT*, *relE* and *relB* genes in BW strain harboring high copy plasmid of *E. coli ybjN* gene compared to the BW strain by qRT-PCR, normalized to the expression value of 16S rRNA (*rrsA*) gene. Cells were grown in M9 medium for 8 h with shaking to an OD_600_ of 0.5–0.8.

To determine whether YbjN affects bacterial growth, optical density and growth rate constants at the exponential phase in BW, *ybjN* mutant and BW over-expressing *ybjN* were determined. As shown in Fig. 2A, both BW and *ybjN* mutant showed a similar growth curves and growth rate constants for BW and the *ybjN* mutant were 2.26 and 2.28, respectively, compared to 1.72 for *ybjN* over-expressing strains grown in LB broth. These results indicate that over-expression of *ybjN* slowed bacterial growth in *E. coli*. When relative expression levels of *ybjN* in BW, *ybjN* mutant, and BW over-expressing *ybjN* in M9 medium were determined by qRT-PCR at mid-logarithmic phase (8 h), it was found that the *ybjN* gene was not expressed in the mutant strain as expected. Multiple copies of *ybjN* resulted in ∼20-fold increase of *ybjN* transcripts in the BW strain (data not shown), suggesting that growth inhibition might also be related to expression levels of *ybjN*.

Recent reports revealed the importance of toxin-antitoxin genes such as *mqsR/ygiT* and *relE/relB* in controlling bacterial growth [18]. To determine whether the inhibitory effect of *ybjN* over-expression on bacterial growth is mediated by or related to toxin-antitoxin genes, qRT-PCR was conducted to quantify the relative expression of *mqsR*, *ygiT*, *relE*, and *relB* in both BW and BW strain over-expressing *ybjN*. Results showed that *mqsR*, *ygiT*, *relE* and *relB* genes in the BW strain over-expressing *ybjN* were significantly up-regulated 15.8, 9.9, 5.6 and 3.1-fold, respectively, as compared to those in the BW strain (Fig. 2B). This indicate that over-expression of *ybjN* in BW strain contributes to an imbalance of toxin and antitoxin genes, suggesting that the inhibitory effect of over-expression of *ybjN* on bacterial growth might be partially related to induction of toxin-antitoxin genes.

### YbjN negatively regulates fimbriae formation and over-expression of ybjN results in autoaggregation

**Figure 3:**
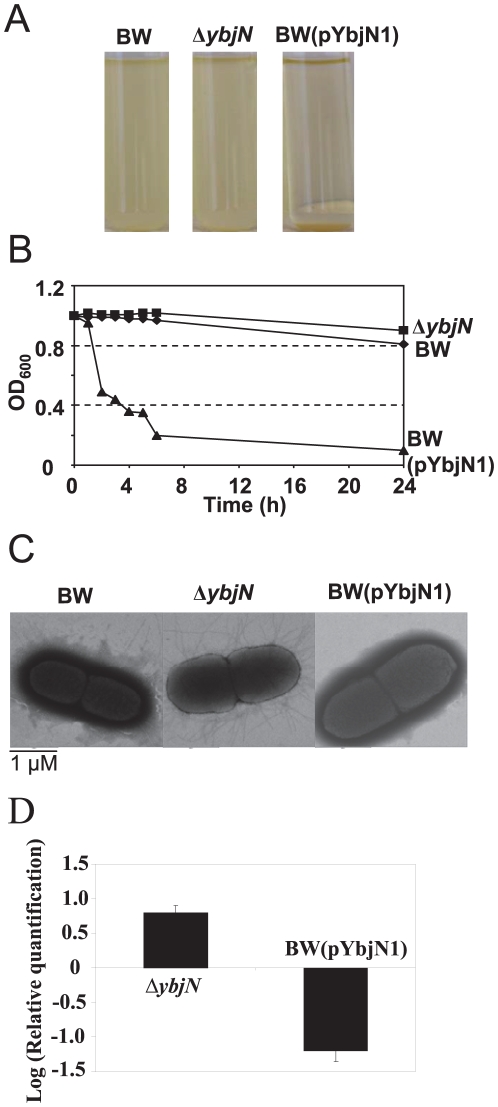
YbjN negatively regulates fimbriae formation and over-expression of *ybjN* results in autoaggregation. A. Demonstration of quick autoaggregative phenotype conferred by *ybjN* overexpression. Cells were grown in LB broth overnight with shaking at 37°C and allowed to settle for 6 h. BW: *E. coli* strain BW25113; BW(pYbjN1): *E. coli* strain BW25113 containing *E. coli ybjN* gene in *pGEM T easy* vector; B. Settling profile for *E. coli* strain BW25113, *ybjN* mutant and *E. coli* strain BW25113 over-expressing *ybjN*. Cells were grown in LB broth overnight at 37°C with shaking. At the beginning of the experiment, all overnight cultures were mixed vigorously by vortexing for 10 s and OD_600_ for each tube was measured. Tubes were then left at room temperature without interference and OD_600_ for each tube was measured at the time points indicated. C. Transmission electron microscopy analysis of fimbriae formation for *E. coli* strain BW25113, *ybjN* mutant and *E. coli* strain BW25113 over-expressing *ybjN*. Bacteria were grown with 250 rpm shaking at 34°C in M9 medium to OD600 = 0.5∼0.8. Cells were negatively stained with 1% phosphotungstic acid (pH 7.0) and micrographs were taken at an accelerating voltage of 80 kV. Pictures were selected from a representative of cells. D. Relative expression of *fimD* gene in *ybjN* mutant and *E. coli* strain BW25113 over-expressing *ybjN* compared to BW strain at OD_600_ = 0.5∼0.8 in M9 medium by qRT-PCR, normalized to the expression value of 16S rRNA (*rrsA*) gene. Bacteria were grown with 250 rpm shaking at 34°C in M9 medium to OD_600_ = 0.5∼0.8.

We observed that bacterial strain over-expressing *ybjN* precipitated within a few hours after removal from a shaker (Fig. 3A). To gain more insight into this autoaggregation phenomenon, the kinetics of YbjN-mediated autoaggregation were investigated. As shown in Figs. 3A and 3B, cells of BW over-expressing *ybjN* rapidly autoaggregated and settled within 6 h when the liquid culture was left undisturbed. In contrast, cells of the BW and *ybjN* mutant in liquid culture retained their planktonic forms under similar conditions (Figs. 3A and 3B). On the other hand, the *ybjN* mutant appeared less likely to aggregate compared to BW (Fig. 3B). These results indicate that over-expression of *ybjN* resulted in autoaggregation.

Previous studies have reported that autoaggregation is inversely correlated with fimbrial formation [19]. Transmission electron microscopy was used to visualize the Type I fimbriae formation for the BW, *ybjN* mutant, and strains over-expressing *ybjN*. Deletion of the *ybjN* gene led to over-production of fimbriae, and the *ybjN* mutant produced about three times more fimbriae than that of the BW (Fig. 3C). In contrast, no pili were observed on cells over-expressing *ybjN*.

To correlate fimbrial formation with fimbrial gene expression, the relative expression of *fimD*, gene encoding an outer membrane usher protein for fimbriae biosynthesis, was quantified in the BW, *ybjN* mutant and strain over-expressing *ybjN* using qRT-PCR (Fig. 3D). The *fimD* gene was up-regulated 8-fold in the *ybjN* mutant, but it was down-regulated 12-fold in strain over-expressing *ybjN* as compared to that of the BW (Fig. 3D). These results indicate that YbjN negatively regulated fimbriae formation by suppressing fimbrial gene expression, suggesting that the observed autoaggregation in the strain over-expressing *ybjN* might be due to deficiency of Type I fimbriae formation.

### YbjN suppresses bacterial motility and negatively regulates flagellar gene expression

**Figure 4:**
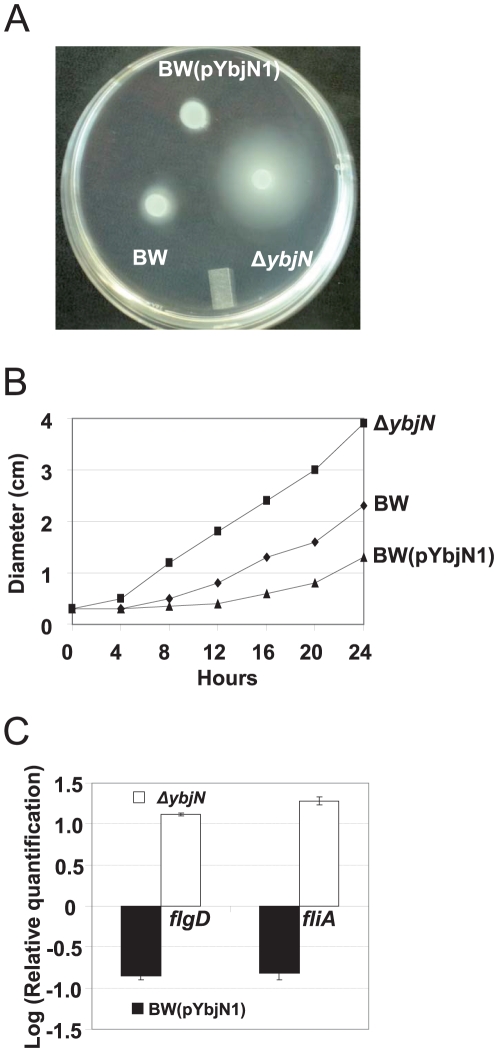
YbjN suppresses bacterial motility and over-expression of *ybjN* negatively regulates flagellar gene expression. A. Migration phenotypes of *E. coli* strain BW25113 (BW), *ybjN* mutant and BW over-expressing *ybjN* at12 h after incubation. B. Comparison of the movement distance of BW, *ybjN* and BW over-expressing *ybjN*. Cells were inoculated on the surface of a motility plate containing 0.25% agar as described previously [20]. Plates were incubated at 30°C for 24 h, during which the displacement (diameter) of the outermost edge of the movement was measured. The experiment was performed for three times in triplicate. Errors bars were smaller than the symbols. C. Relative expression of *fliA* and *flgD* gene expression in *ybjN* mutant and BW over-expressing *ybjN* compared to BW strain. Bacterial strains were grown overnight in LB broth and re-inoculated in 5 mL M9 medium. Relative expression of *fliA* and *flgD* genes, normalized to the expression value of 16S rRNA (*rrsA*) gene, was determined by qRT-PCR after 8 h growth (OD_600_ at 0.5–0.8) in M9 medium.

Bacterial motility was assessed for the BW, *ybjN* mutant and BW strain over-expressing *ybjN* by inoculating bacterial cells on a motility plate (0.25% agar) and measuring the diameter of the circle covered by bacterial cells for up to 24 h as described previously [20]. The *ybjN* mutant exhibited enhanced motility compared with that of the BW strain 12 h following inoculation (Figs. 4A and 4B). In contrast, BW strain over-expressing *ybjN* did not move in the first 12 h. Diameters of circles of the BW, *ybjN* mutant, and strain over-expressing *ybjN* at 12 h were about 0.84±0.02, 1.88±0.04, and 0.41±0.01 (longest part of the irregular movement), respectively. These results suggest that the YbjN is a negative regulator of bacterial motility in *E. coli*.

qRT-PCR was utilized to determine flagellar-related gene expression in these bacterial strains, including *fliA* (sigma F factor) and *flgD* (flagellar hook assembly protein), in M9 medium at mid-logarithmic phase. Expression of *fliA* and *flgD* in the *ybjN* mutant was up-regulated by 12.5 and 11.3 fold, as compared to that of the BW (Fig. 4C). Whereas over-expression of *ybjN* in the BW resulted in 8.8 and 9.2 fold lower levels of expression of *fliA* and *flgD* genes, respectively. These findings indicate that YbjN negatively regulates flagellar gene expression, and this suppression is correlated with expression levels of the *ybjN* gene.

### YbjN suppresses capsular biosynthesis

**Figure 5:**
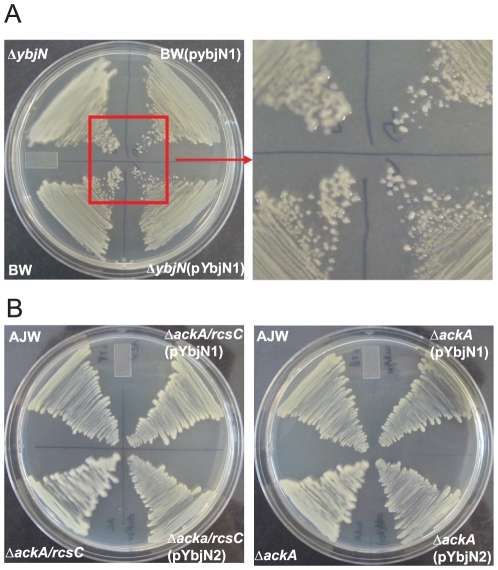
Impact of *ybjN* on capsule biosynthesis. A. *ybjN* negatively regulates colanic acid biosynthesis. Growth of *E. coli* strain BW25113 (BW), *ybjN* mutant and BW over-expressing *ybjN* on LB plates supplemented with 700 mM NaCl at 37°C, showing mucoid phenotype of *ybjN* mutant. B. Over-expression of *ybjN* from both *E. coli* and *E. amylovora* suppresses mucoid phenotypes of *E. coli* strain AJW678, *ackA* and *ackA/rcsC* mutants. Bacteria were grown in LB for 24 h at 28°C with shaking. 10 µl bacterial cells were streaked on LB plates supplemented with 700 mM NaCl and incubated at 28°C as described previously [20]. Pictures were taken at 24 h.

To determine whether YbjN also influences capsular biosynthesis, levels of mucoidy of the BW, *ybjN* mutant, and strains over-expressing *ybjN* were compared after incubation for 24 h at 37°C on LB plate containing 700 mM sodium chloride. As shown in Fig. 5A, colonies of the *ybjN* mutant exhibited a highly mucoid phenotype, characteristic of cells over-producing colanic acid. BW colonies also appeared mucoid, although to a lesser extent than that of the *ybjN* mutant (Fig. 5A). In contrast, non-mucoid colonies were observed for both BW and *ybjN* mutant strains harboring multiple copies of *ybjN* plasmids from both *E. coli* and *E. amylovora*. These observations indicate that YbjN negatively regulates capsule formation.

To further explore how YbjN affects capsule biosynthesis, a multicopy *ybjN* plasmid was introduced into *E. coli* strain AJW678 (AJW), *ΔackA* and *ΔackA/rcsC* mutant strains. These mutant strains are highly mucoid due to over-expression of acetyl∼P, and hence phospho-RcsB as reported previously [20]. As reported, *ackA* and *ackA/rcsC* mutants formed mucoid colonies after 24 h of incubation on LB plates (Fig. 5B, Frederick *et al.*, 2006). Interestingly, multiple copies of *E. coli* and *E. amylovora ybjN* suppressed the mucoid phenotype in both *ackA* and *ackA/rcsC* mutants, and the colonies were similar to those of the AJW strain (Fig. 5B). These results indicate that YbjN is a negative regulator of capsule biosynthesis, suggesting that YbjN might directly affect phosphorylation of RcsB in the Rcs phosphorelay system.

### Negative impact of ybjN on biofilm formation

**Figure 6:**
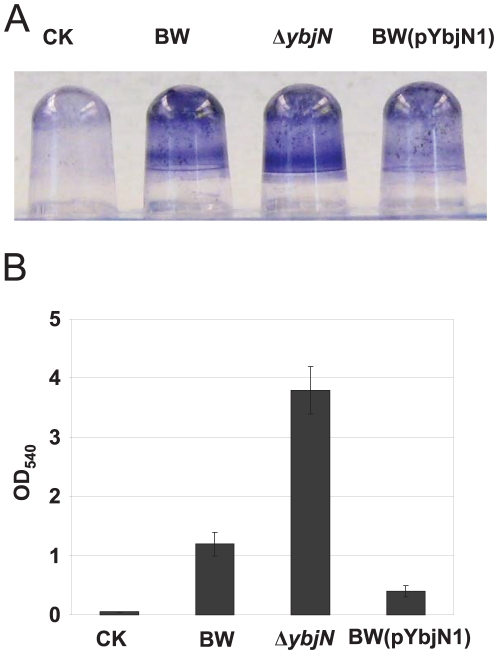
YbjN is a negative regulator of biofilm formation. A. All strains were grown overnight in LB broth at 30°C, diluted (1∶150) in LB and incubated at 30°C, without shaking, in PVC plates. After 48 h, the extent of biofilm formation was assessed by staining the wells with 1% crystal violet (CV). B. Quantification of CV staining. The amount of CV staining (and therefore biofilm formation) was quantified at OD_540_. CK: negative water control.

Previous studies have indicated that production of colanic acid in *E. coli* K-12 is important for maturation of biofilms [21]. On the other hand, fimbriae and flagella are required for initial attachment during biofilm formation in *E. coli* [22]. Therefore, we hypothesized that YbjN may also play a role in biofilm formation. To prove this hypothesis, the abilities of the BW, *ybjN* mutant and strain over-expressing *ybjN* to form biofilms on a polyvinyl chloride (PVC) microtitre plate were evaluated as previously described (Ferrières and Clarke, 2003). As expected, the crystal violet (CV) staining for BW was obvious compared to the control, indicating that the BW strain forms a good biofilm on a PVC surface (Fig. 6A). The amount of biofilm formed by the *ybjN* mutant was much more than that of BW strain, whereas biofilm formation of BW strain with multi-copy plasmids containing *ybjN* was greatly impaired (Fig. 6A). Quantitative analysis revealed that biofilms formed by the *ybjN* mutant and strain over-expressing *ybjN* were about 3 fold higher than, or half of that of the BW strain, respectively (Fig. 6B). These results indicate that YbjN suppresses normal biofilm formation in *E. coli*.

### Over-expression of ybjN confers acid susceptibility

**Figure 7:**
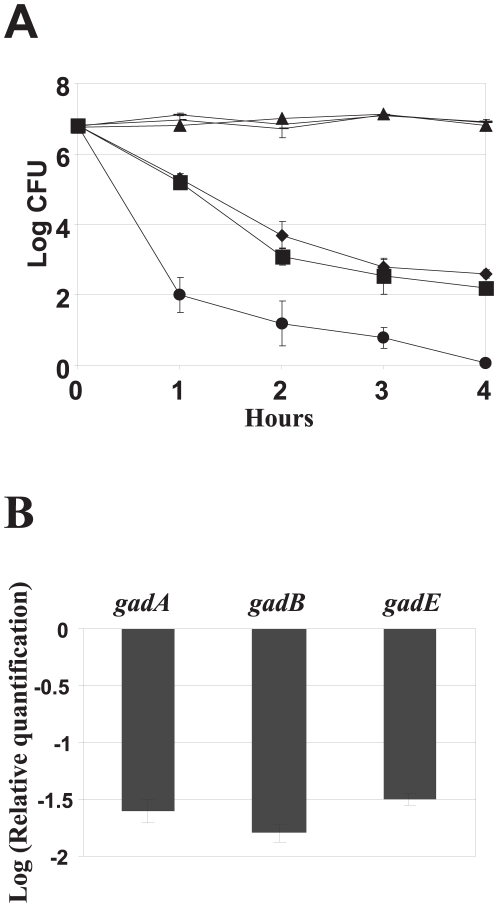
Over-expression of *ybjN* confers acid susceptibility. A. Effect of *ybjN* on acid susceptibility. Cells were grown for 18 h in M9 medium and bacterial suspensions were incubated in EG medium at pH 2.5 as described previously [44]. Surviving bacterial cells were counted at time 0, 1, 2, 3 and 4 h by dilution plating on LB plates.▴: *E. coli* strain BW25113 (BW), pH 7.0; _: *ybjN*, pH 7.0; -: BW (pYbjN1), pH 7.0; ▪: BW, pH 2.5, ♦: *ybjN*, pH 2.5, •: BW (pYbjN1), pH 2.5. B. Relative expression of acid resistance genes in *ybjN* over-expression strain. Bacterial strains were grown overnight in LB broth and re-inoculated in 5 mL M9 medium. Cells were grown in M9 medium for 8 h with shaking to an OD_600_ of 0.5–0.8. Relative expression of *gadA*, *gadB* and *gadE* genes in *ybjN* over-expression strain compared to BW strain was determined by qRT- PCR, normalized to the expression value of 16S rRNA (*rrsA*) gene.

Commonly, many enteric bacteria encounter extreme acidic pH conditions when interacting with their host, such as traveling through the gastrointestinal tract. Based on this observation, we investigated whether YbjN confers acid resistance. *E. coli* BW, *ybjN* mutant and strain over-expressing *ybjN* were evaluated for their survival at pH 2.5 and 7. All three strains exhibited similar survival rate at pH 7.0 (Fig. 7A). When subjected to strong acidic conditions, both the BW strain and the *ybjN* mutant showed slight resistance to acid conditions than that of the strain over-expressing *ybjN* (Fig. 7A). Interestingly, the strain over-expressing *ybjN* was highly susceptible to acidic pH (Fig. 7A). After 1 h treatment in pH 2.5, the Log CFU number decreased from 6.8 to 2.0 and by 4 h, no bacterial colonies were formed on the LB plates. The results clearly indicate that over-expressing of *ybjN* leads to increased acid susceptibility.

Previous studies have reported that many genes in the *E. coli* genome are indispensable for bacterial survival under acidic stress conditions including the *gad* operon (glutamate-dependent acid resistance pathway). To determine whether *ybjN*-mediated acid susceptibility is due to changes in gene expression of the *gad* operon, the relative expression of *gadA*, *gadB* and *gadE* grown in M9 medium at mid-logarithmic phase were measured by qRT-PCR. All three genes were significantly down-regulated for about 12 to18 fold in the strain over-expressing *ybjN* as compared to that of the BW strain (Fig. 7B) and the *ybjN* mutant (date not shown). These results suggest that acid susceptibility in *ybjN* over-expression strains might be attributed to suppression of the acid resistance gene expression, including *gadABE*.

### Expression of ybjN is growth stage- and temperature-dependent

**Figure 8:**
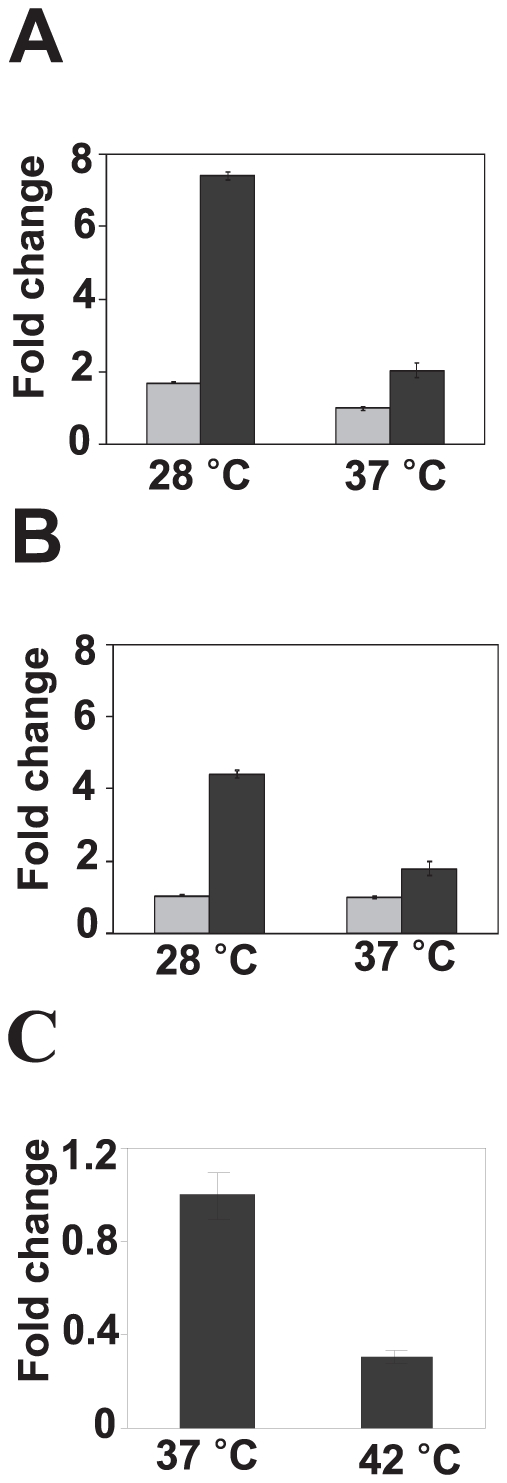
Expression of *ybjN* gene in *E. coli* strain BW25113 strain. A and B. Relative expression level of *ybjN* in LB broth (A) and M9 medium (B). Dark bar: log phase, Light bar: stationary phase. The expression of *ybjN* was measured by qRT-PCR, normalized to the expression value of 16S rRNA (*rrsA*) gene and then compared to *ybjN* expression level at stationary phase at 37°C in M9 medium. Bacterial cells were collected in LB or M9 medium at an OD_600_ at 0.8∼1.0 for log phase and 1.8∼2.1 for stationary phase. C. Relative expression level of *ybjN* at high temperature. Bacterial cells were grown to log phase in M9 at 37°C and then incubated with shaking at 37°C or 42°C for 6 h. The relative expression of *ybjN* at 42°C was measured by qRT-PCR, normalized to the expression value of *rrsA* gene and then compared to the *ybjN* expression level at 37°C.

As indicated above, *ybjN* is a stress-related gene; therefore, expression of the *ybjN* gene in the BW strain under different growth stages and stress conditions were determined. Expression levels of *ybjN* were higher in the log phase than that of the stationary phase in both LB and M9 media (Figs. 8A and 8B). The expression level of *ybjN* decreased when the temperature shifted from 28°C to 42°C (Figs. 8ABC). Transcripts of *ybjN* at 42°C were about 3-fold lower than those at 37°C. These results have demonstrated that expression of *ybjN* is growth stage-, and temperature-dependent.

### Global gene expression profiles in the ybjN mutant and the ybjN over-expression strain

Given the fact that YbjN exerted pleiotropic effects on *E. coli*, we utilized a microarray analysis to globally identify genes regulated by *ybjN* in BW. We compared BW to both the *ybjN* knockout mutant and the *ybjN* over-expression strain grown in M9 medium at 8 h, and found that 47 and 74 genes exhibited increased and decreased transcript abundance in the *ybjN* mutant relative to BW strain, respectively (Table S2). A larger set of genes appeared to be affected by *ybjN* over-expression, with 216 and 293 showed increased and decreased transcript abundance relative to BW strain, respectively (Table S2). Among 39 commonly regulated genes, only nine and two genes were either down- or up- regulated in both the *ybjN* mutant and *ybjN* over-expression strains; whereas, 13 genes were up-regulated in the *ybjN* mutant, but down-regulated in the *ybjN* over-expression strain. In contrast, 15 genes were up-regulated in the *ybjN* over-expression strain, but down-regulated in the *ybjN* mutant. This indicated that deletion and over-expression of the *ybjN* gene resulted in different gene profiles.

### Inverse correlation of ybjN expression with expression of flagellar, fimbrial and acid resistance genes

Consistent with our qPCR results, the *fliA* gene was up-regulated 16-fold in the *ybjN* mutant and down-regulated 3.6-fold in the *ybjN* over-expression strain (Table 1). Several FliA controlled genes including *flgB*, *flgD*, *flgE*, *fliL* and *fliM* were up-regulated in the *ybjN* mutant, but down-regulated in the *ybjN* over-expression strains (Table 1 and Table S2). These results further demonstrate that the *ybjN* negatively regulates bacterial motility by affecting flagellar gene expression. *ybjN* expression was also inversely correlated with expression of fimbrial genes. Genes encoding major components of bacterial pili including *fimC*, *fimD* and *fimG* were significantly up-regulated in the *ybjN* mutant, but down-regulated in the *ybjN* over-expression strain (Table 1 and Table S2). Interestingly, curli genes (*crl* and *csgDEFG*) were significantly down-regulated in the *ybjN* mutant, but only slightly up-regulated in the *ybjN* over-expression strain (Table S2). These results suggest that YbjN plays an important role in the regulation of bacterial surface appendages.

**Table 1:** Flagellar, fimbrial, and acid resistance genes that are inversely regulated by *ybjN* (p-value<0.05).

Gene Name	Gene Function	*ΔybjN*/BW	*ybjN*_Over-expression/BW
*ybjN*	Putative sensory transduction regulator	0.02	18
Flagellar genes*			
*flgB*	flagellar component of cell-proximal portion of basal-body rod	16.57	0.25
*flgD*	flagellar hook assembly protein	15.27	0.28
*flgE*	flagellar hook protein	12.62	0.31
*fliA*	RNA polymerase, sigma 28 (sigma F) factor	16.22	0.28
*fliL*	flagellar biosynthesis protein	7.39	0.29
*fliM*	flagellar motor switching and energizing component	7.48	0.33
Fimbrial genes*			
*fimC*	chaperone, periplasmic	2.31	0.41
*fimD*	outer membrane usher protein, type 1 fimbrial synthesis	3.06	0.55
*fimG*	minor component of type 1 fimbriae	2.27	0.48
Acid resistance genes*			
*gadA*	glutamate decarboxylase A	2.55	0.05
*gadB*	glutamate decarboxylase B	2.93	0.08
*gadC*	predicted glutamate∶gamma-aminobutyric acid antiporter	2.63**	0.19
*gadE*	Transcriptional regulator	1.62**	0.12
*gadW*	Transcritional regulator	0.84**	0.28
*gltD*	Glutamate synthase	1.078**	0.34
*gltF*	Regulator of gltBDF operon	0.907**	0.32
*slp*	Membrane protein	1.88**	0.13
*evgA*	Response regulator	0.68**	0.45
*evgS*	Sensor kinase	0.64**	0.24
*hdeA*	Acid stress gene	2.01**	0.09
*hdeB*	Acid stress chaperon	1.69**	0.05
*hdeD*	acid-resistance membrane protein	2.68	0.10

*Selected genes with p-value<0.05 were listed; Others see Table S1.

**p>0.05. BW: *E. coli* K-12 strain BW25113.

In addition, acid resistant-related genes including the general acid resistance genes (*hdeABD*) as well as glutamate-dependent acid resistance genes (*gadABC*) were also inversely affected in the *ybjN* mutant and *ybjN* over-expression strains (Table 1 and Table S2). Furthermore, the *evgA* response regulator and the *gadE* transcription activator, both regulators for *gadABC*, were down-regulated by 2 to 9 fold, respectively, in the *ybjN* over-expression strain (Table S2). These results indicate that *ybjN* over-expression might shut down the glutamate-dependent acid resistance pathway, leading to a highly acid susceptibility phenotype as described above.

Other groups of genes that were inversely affected in the *ybjN* mutant and *ybjN* over-expression strain include genes involved in fructose, maltose, and nitrite metabolism and export, such as *malEFGKMT* and *lamB*, *narK*, *nirBD*, *nac*, *napABGH*, and *glnAGKL* (Table 1 and Table S2).

### Over-expression of ybjN leads to down-regulation of metabolic pathways and up-regulation of stress-related genes

Among up-regulated genes including many hypothetical genes, many stress-related genes were expressed at higher levels in *ybjN* over-expression strains that in the BW (Table 2, Table S2), These included cold shock (*cspABG*), the SOS stress response (*recA*, *sulA*, *uvrB*, and *umuD*), several toxin/antitoxin (*mqsRygiT*, *relBE*, and *rhpSB*), and transporter genes (*betT*, *pstABCS*, *ssuCDE* and *tauABC*). This suggests that over-expression of *ybjN* confers higher fitness under stress conditions.

**Table 2:** Stress responsive genes that are induced by *ybjN* over-expression (p-value<0.05).

Gene Name	Gene Function	*ybjN*_over-expression/BW
Cold shock genes		
*cspA*	major cold shock protein	3.00
*cspB*	Qin prophage; cold shock protein	8.83
*cspG*	DNA-binding transcriptional regulator	12.44
SOS stress response genes		
*sulA*	SOS cell division inhibitor	6.96
*umuD*	SOS mutagenesis; error-prone repair; processed to UmuD; forms complex with UmuC	2.79
*recA*	DNA strand exchange and renaturation, DNA-dependent ATPase,	2.24
*recN*	protein used in recombination and DNA repair	4.46
*recX*	regulator, OraA protein	3.06
*uvrB*	DNA repair; excision nuclease subunit B	2.08
Toxin-Antitoxin		
*mqsR*	predicted cyanide hydratase	12.57
*ygiT*	predicted DNA-binding transcriptional regulator	9.77
*relB*	Qin prophage; bifunctional antitoxin of the RelE-RelB toxin-antitoxin system/transcriptional repressor	3.47
*relE*	toxin of the RelE-RelB toxin-antitoxin system	5.00
*chpS*	antitoxin of the ChpB-ChpS toxin-antitoxin system	3.18
Transporter genes		
*betT*	high-affinity choline transport	3.88
*pstA*	high-affinity phosphate-specific transport system	3.50
*pstB*	ATP-binding component of high-affinity phosphate-specific transport system	2.73
*pstC*	high-affinity phosphate-specific transport system, cytoplasmic membrane component	2.93
*pstS*	high-affinity phosphate-specific transport system; periplasmic phosphate-binding protein	4.57
*ssuC*	alkanesulfonate transporter subunit	4.20
*ssuD*	alkanesulfonate monooxygenase, FMNH(2)-dependent	3.29
*ssuE*	NAD(P)H-dependent FMN reductase	5.47
*tauA*	taurine transport system periplasmic protein	6.22
*tauB*	taurine ATP-binding component of a transport system	3.29
*tauC*	taurine transport system permease protein	4.45

In contrast, metabolic pathway genes were down-regulated in *ybjN* over-expression strain, including the glyoxylate shunt (*aceABK*), the tricarboxylic acid (TCA) cycle (*fumC*), glycolysis (*gapA*, *gpmM*, *eno*) and oxidative phosphorylation (*cyoABCDE*, *cydAB*, *atpDF* and *ccmDFGH*) (Table 3, Table S2). Consistent with growth inhibitory effects, biosynthetic genes including biotin (*bioABCDF*); amino acids (*asnAC*, *hisACDGI*, *glnA*, *gdhA*, *gltDF*, *metACEH*, *ilvCHI*, and *trpACDE*) and nucleotide biosynthesis (*guaAB*, *pntAB*, *purBCDEFHKLMNT* and *pyrBCDF*) were also significantly down-regulated (Table S2 and Table S3). Interestingly, many heat shock genes (*groSL*, *clpB* and *htpG*) were also down-regulated in *ybjN* over-expression strains (Table S2). These results suggest that *ybjN* over-production might lead to slow growth due to metabolic slow-down, thus resulting in a phenotypic switch into a persistent state.

**Table 3:** Carbohydrate catabolism genes that are suppressed in *ybjN* over-expression strain (p-value<0.05).

Gene Name	Gene Function	*ybjN*_over-expression/BW
Glyoxylate shut		
*aceA*	isocitrate lyase	0.43
*aceB*	malate synthase A	0.35
*aceK*	isocitrate dehydrogenase kinase/phosphatase	0.38
Glycolysis		
*gapA*	glyceraldehyde-3-phosphate dehydrogenase A	0.30
*gpmM*	phosphoglycero mutase III, cofactor-independent	0.35
*eno*	enolase	0.38
TCA cycle		
*fumC*	Fumarate hydratase class II	0.35
Oxidative Phosphorylation		
*cyoE*	protoheme IX farnesyltransferase	0.34
*cyoD*	cytochrome o ubiquinol oxidase subunit IV	0.33
*cyoC*	cytochrome o ubiquinol oxidase subunit III	0.35
*cyoB*	cytochrome o ubiquinol oxidase subunit I	0.39
*cyoA*	cytochrome o ubiquinol oxidase subunit II	0.43
*cydA*	cytochrome d terminal oxidase, polypeptide subunit I	0.26
*cydB*	cytochrome d terminal oxidase polypeptide subunit II	0.29
*atpD*	membrane-bound ATP synthase, F1 sector, beta-subunit	0.48
*atpF*	membrane-bound ATP synthase, F0 sector, subunit β	0.47
*ccmD*	heme exporter protein C	0.48
*ccmF*	cytochrome c-type biogenesis protein	0.25
*ccmG*	disulfide oxidoreductase	0.41
*ccmH*	possible subunit of heme lyase	0.45

## Discussion

Understanding the functions of unknown genes, which typically account for about half of those present in a particular genome, is one of the great challenges facing biologists in the post genomic era. These unknown or hypothetical genes become major barriers in understanding the basic biology of organisms. In this study, we attempted to characterize one such hypothetical orphan gene, *ybjN*, in *E. coli*. Among our findings, it was revealed that *ybjN* expression correlated with its ability to rescue the temperature sensitivity of the ts9 mutant strain. Moreover, we demonstrated that both *ybjN* knockout and over-expression resulted in pleiotropic effects on bacterial growth, swarming motility, fimbriation, capsule formation, biofilm formation and acid resistance. These phenotypic observations were further confirmed by global gene expression analysis using an *E. coli* microarray. Most strikingly, over-production of *ybjN* significantly induced many stress response genes, but suppressed genes involved in multicellular behavior, energy metabolism and biosynthesis, suggesting that YbjN might play a significant role in survival under certain stress conditions. Furthermore, our results indicate that the YbjN homologs from different enterobacteria are functionally conserved, thus suggesting evolutionary conservation for this orphan protein.

Previously, Chen *et al.*, 2006 have reported that multiple copies of *ybjN* suppress temperature sensitivity of the ts9 mutant [8]. In this study, results reconfirmed this finding and further demonstrated that expression levels of the *ybjN* gene decreased by 10-fold in the ts9 mutant, whereas multiple copies of the *ybjN* gene resulted in a 100-fold increase of *ybjN* transcripts in the ts9 strain, strongly indicating that over-expression of *ybjN* transcripts might lead to rescue of the temperature sensitive phenotype of *E. coli*. Furthermore, the ts9 mutant containing multiple copies of the *ybjN* gene could not grow at 40°C, at which temperature most ts9 spontaneous revertants are capable of growing, indicating that the rescued strain is not the result of reversion of the point mutation [8]. Upon sequencing the *coaA* gene from several colonies (data not shown), there was no evidence of any reversion of the point mutation.

Our data have further revealed that expression of *ybjN* at 42°C is significantly lower than that at 37°C, further suggesting that rescue of temperature sensitivity of the ts9 strain may indeed be directly related to levels of expression of the *ybjN*. However, although the *ybjN* expression levels are positively correlated to ability of the ts9 mutant to survive at high temperatures, the mutation of the *ybjN* itself did not result in temperature sensitivity, and growth of the *ybjN* mutant is similar to that of the BW strain (data not shown). These results indicate that decreased *ybjN* expression in the ts9 mutant alone may not directly contribute to its temperature sensitivity, but over-expression of *ybjN* is required for its ability to grow at higher temperature. We still do not know why mutations in the ts9 strain result in the reductions in *ybjN* expression levels.

Our microarray results may have shed some light on solving the mysteries of the above mentioned phenomenon. We demonstrated that over-expression of *ybjN* led to significant down-regulation of genes involved in metabolism, but promotion of stress-related gene expression such as the toxin-antitoxin modules, the SOS stress response, cold shock and phage-shock genes. This expression pattern is very similar to those reported for so-called persister cells [23]. Persister cells are dormant variants of normal bacterial cells and are highly tolerant to antibiotics [24]. Transcriptome studies of persister cells have revealed down-regulation of biosynthesis genes and increased expression of toxin/antitoxin modules (MqsRYgiT, RelBE, MazEF, DinJYafQ, HipBA), SOS response and cold shock genes [25]. A recent study reported that MqsR promotes formation of persister cells through activation of the cold shock protein CpsD ([26a] [26b]). It has also been reported that over-expression of the RelE toxin, an inhibitor of translation, results in cellular function shut-down and a sharp increase in persisters [27]. On the contrary, deletion of the *hipA* gene decreases in persister formation in both stationary and biofilm populations [28]. In this study, *ybjN* over-expression strains may also induce a dormancy state similar to that of persister cells, which leads to growth slowdown (inhibition) as well as phenotypic switch to a state similar to that of persistence [29].

Several independent studies have revealed that autoaggregation has an inverse correlation with flagellar and fimbriae production [19], [30]. Expression of autoaggregation factor antigen 43 and genes involved in flagellation and fimbriation are mutually exclusive. Moreover, autoaggregation strains caused by overproduction of antigen 43 are deficient in bacterial appendage production. In this study, it has been demonstrated that *ybjN* over-expression mediated-autoaggregation down-regulated flagellar and fimbrial gene expression, hence resulting in reduced motility and fimbriation. In contrast, a mutation in *ybjN* led to over production of flagella and fimbriae, thus increasing motility and fimbriation, and indicating that YbjN is a negative regulator of flagellar and fimbriae biosynthesis. However, antigen 43 is not up-regulated in the *ybjN* over-expression strain, suggesting that YbjN's effect on autoaggregation is independent of the antigen 43 pathway. Aggregation factors mediating cell clumping provide protection against phagocytosis and host defense response. Indeed, expression of the *ybjN* was up-regulated about 3-fold in *E. coli* O157:H7 following human macrophage infection [15]. These results indicate that *ybjN* over-expression might eliminate bacterial surface structures to promote autoaggregation and elude host defense responses during host colonization. Indeed, one quorum sensing gene *sdiA* is significantly up-regulated in strain over expressing *ybjN* (Table S2), thus resulting in changes in multi-cellular behavior.

It has been well-documented that both cell appendages and colanic acid contribute to the normal biofilm formation in *E. coli*. Cell appendages such as pili and flagella are required for bacterial attachment to surfaces and initiation of biofilm formation [22], [31]. On the other hand, colanic acid production is required for the maturation of biofilms [21]. In this study, the *ybjN* mutant has exhibited increased colanic acid biosynthesis and pili formation; whereas, strain over-expressing *ybjN* was highly reduced in both colanic acid production and pili formation. The impact of *ybjN* on pili and capsule formation is correlated with biofilm formation in *E. coli* (Fig. 6). These findings suggest that it is likely that *ybjN* over-expression strains are unable to effectively attach themselves to plate surfaces. Overall, these results clearly indicate that *ybjN*, as a suppressor, may play an important role in regulating multi-cellular behavior, that are essential for bacteria to transit to different life styles and survival.

Expression of exopolysaccharide biosynthetic genes in both *E. coli* and *E. amylovora* is directly controlled by the Rcs phosphorelay system [32], [33], [34]. Mutation of *rcsC* leads to overproduction of exopolysaccharide [32], [33]. The heterodimer formed by the response regulator RcsB and the accessary protein RcsA binds to the promoter of the exopolysaccharide biosynthetic genes and activate gene expression [34]. The *ackA* and *ackA/rcsC* mutants are also mucoid due to the accumulation of acetyl phosphate, which results in increased phosphorylation of RcsB [20]. We previously reported that as a homolog of *E. coli ybjN*, the *E. amylovora ybjN* mutant exhibits over-production of amylovoran, similar to that of the *ybjN* mutant in this study [16]. On the other hand, over-expression of *Erwinia ybjN* greatly suppresses amylovoran in various amylovoran-over-producing mutant strains such as *rcsC*, *envZ*, *ompR*, *grrA*, *grrS* and *hns* mutants (Wang and Zhao, unpublished data). Similarly, over-expression of *ybjN* in the *ackA* and *ackA/rcsC* mutants strongly suppresses the mucoid phenotypes in *E. coli*. It is possible that YbjN interacts with RcsB to affect its phosphorylation or RcsA to decrease DNA binding activities. YbjN may also directly bind to the promoter as a suppressor. However, our microarray results suggest that, the most likely scenario may be that *ybjN* over-expression contributes to extremely low metabolic activity within the bacterial cell, resulting in depletion of substrates for bacteria to synthesize copious amount of colanic acid. These results further confirm the negative regulatory effect of *ybjN* on exopolysaccharide production. Further studies are needed to determine the mechanism as to how YbjN interferes with exopolysaccharide production.

*E. coli* has a higher level of acid resistance than that of other characterized enterobacteria, mainly due to the presence of many unique acid resistance mechanisms that are absent in other genera [35], [36]. Among them, the GadE transcriptional regulator is critical for activation of the glutamate-dependent acid resistance pathway. The GadE controls the expression of two glutamate decarboxylase GadAB, the glutamate antiporter GadC, two glutamate synthase GltBD, the acid stress chaperones HdeAB and the membrane proteins HdeD and Slp. GadE, a LuxR family regulator, is controlled by two AraC-like regulators, GadX and GadW as well as the EvgAS two-component system [36], [37], [38]. It has been reported that over-expression of *gadE* confers acid resistance by activating GadABC [39]. In addition, within the acid fitness island, the HdeABD operon increases acid resistance by preventing formation of aggregation due to acid-denatured proteins in the periplasmic space [40]. Interestingly in this study, our microarray data demonstrate that all of the above mentioned genes were significantly down-regulated in the *ybjN* over-expression strain (Table 1), which is highly susceptible to acid conditions, thus indicating that YbjN is a negative regulator of acid resistance in enteric bacteria by suppressing the glutamate-dependent acid resistance pathway.

As an emerging concept in microbial research, multicellular behaviors of bacterial populations may be related to bacterial subpopulation programmed cell death, which may preserve metabolic resources and avoid host immune response. One such genetic cause of subpopulation cell death is mediated by the toxin-antitoxin modules such as *mazEF* [41]. In *E. coli*, toxin-antitoxin modules are stress-induced suicide modules that trigger cell death. In this study, it is clear that toxin-antitoxin modules (RelBE and ChpBS and MqsR/ygiT) were highly induced along with suppression of metabolism in the *ybjN* over-expressing strain, suggesting YbjN may be involved in modulating programmed cell death, thus preventing host recognition. Indeed, whole-genome expression profiling has revealed that *ybjN* is significantly induced in *E. coli* during growth on mucus, conditions designed to mimic the human intestine [14]. Expression of the *ybjN* gene is also increased by 3-fold in enterohemorrhagic *E. coli* O157:H7 following human macrophage infection [15]. This data suggests that YbjN may play a significant role in bacterial virulence by modulating cell death. More studies are needed to test this hypothesis.

Though our data have clearly demonstrated that YbjN plays important roles in regulating bacterial multicellular behavior, metabolism and survival under stress conditions in *E. coli*, we still do not precisely understand what are the molecular mechanisms underlying these observations. In our future studies, we plan to focus on determining its biochemical function and molecular structure of YbjN protein. Previous genome-wide pull-down screening has not identified any YbjN-interacting proteins in *E. coli*, suggesting that YbjN may not function in protein-protein interactions or the interactions may be transient if there are any [9]. N-terminal His-tag fusion of YbjN protein can be purified from soluble lysate of *E. coli*, suggesting YbjN may be a soluble protein (Zhao, unpublished data). The deduced amino acid sequences of YbjN proteins contain high number of aromatic residues (13 residues for *E. coli* and 12 residues for *Erwinia*), indicating the YbjN proteins also contain the hydrophobic regions, which may be important for membrane association or protein-protein interactions [42], [43]. In addition, it is possible that YbjN may act as a DNA-binding protein, as we have shown that it regulates gene expression. Understanding the biochemical function and structure of YbjN protein will be an important step towards fully elucidating the roles of YbjN in *E. coli* and other enterobacteria.

## Materials and Methods

### Bacterial strains and media

Bacterial strains and plasmids used in this study are listed in Table S4. The LB broth and M9 medium [20] are used routinely for culturing *E. coli*. Acid resistance assay was carried out in minimal E medium containing 0.4% glucose (EG: MgSO_4_-7H_2_O (0.2 g), citric acid-H_2_O (2 g), K_2_HPO_4_.anhydrous (10 g), and NaNH,HPO_4_-4H_2_O (0.35 g) in 1 L distilled water) [44]. When necessary, the following antibiotics, 50 µg ml^−1^ kanamycin and 100 µg ml^−1^ ampicillin, were added to the medium.

### DNA manipulation and sequence analysis

Plasmid DNA purification, PCR amplification of genes, isolation of fragments from agarose gels, cloning, and restriction enzyme digestion and T4 DNA ligation were performed using standard molecular procedures [45]. DNA sequencing was performed at the Keck Center for Functional and Comparative Genomics at the University of Illinois at Urbana-Champaign. Sequence management and contig assembly were conducted using Sequencher 4.9 software. Database searches were conducted using the BLAST programs at NCBI (www.ncbi.nlm.nih.gov/BLAST) [17].

### Cloning ybjN gene from E. coli and E. amylovora

For *ybjN* over-expression, flanking sequences of the *ybjN* ORF in *E. coli* and *E. amylovora* were used to design primers to amplify fragments of the genes and their promoter sequences. Primer pairs ybjN1–ybjN2 without restriction sites and ybjN3–ybjN4 were used to amplify 0.9 kb and 1.1 kb DNA fragments from *E. coli* K12 and *E. amylovora* Ea1189 strain, containing upstream and downstream sequences of the *ybjN* gene, respectively. The two PCR fragments were cloned into pGEM T-easy vector through A-T ligation. The final plasmids were designated as pYbjN1 and pYbjN2, respectively, and their genotypes were confirmed by sequencing. All plasmids were introduced into *E. coli* strain by electroporation. Transformants were selected on LB plates supplemented with ampicillin.

### Growth rate assay

Overnight cultures were inoculated in LB or M9 with the initial OD_600_ at 0.001. The optical density was measured periodically. Growth rate constant was calculate: μ = ((log_10_ N_t_−log_10_ N_0_) 2.303)/(t−t_0_), N_t_: cell number at end point (t) of log phase, N_0_: cell number at beginning (t_0_) of log phase. Bacterial numbers at time points t and t_0_ were determined by dilution plating on LB plates with or without antibiotic.

### Suspension autoaggregation assay

To assess autoaggregation property in different strains, the suspension clumping assay was used to monitor bacterial settling over time as described previously [30]. Overnight bacterial suspensions were grown in LB broth, harvested by centrifugation, and resuspended in 1 ml fresh LB. The concentration for each strain was adjusted to OD_600_ at 1.0 in a 15 ml glass tube. Fifty microliter samples (*n* = 3) were taken approximately 0.5 cm below the surface of the liquid cultures at 0, 1, 2, 3, 4, 5, 6 and 24 h, diluted 10–100 times and then OD_600_ was measured. Data was shown as the mean absorbance ± SD, where the degree of autoaggregation is inversely proportional to the turbidity.

### Visualization of pili by transmission electron microscopy (TEM)

TEM was used to detect fimbriae on bacteria grown in M9 medium. Cells were grown aerobically with 250 rpm agitation at 34°C in M9 to an OD_600_ = 0.8. A 10 µl bacterial cell suspension was adsorbed onto a glow-discharged Formvar-coated copper grid for 10 min. Samples were negatively stained with 1% phosphotungstic acid (pH 7.0) and after 1 min the grid was washed carefully in sterile distilled water and visualized on a Philips CM200 electron microscope (FEI Company). Micrographs were taken at an accelerating voltage of 80 kV.

### Bacterial motility assay

For *E. coli* WT, *ybjN* mutant, and *ybjN* over-expression strain, bacterial cell suspensions were grown overnight in LB broth. Five µl of the bacterial suspension was plated onto the center of motility agar plates (10 g tryptone, 5 g NaCl, 2.5 g agar per liter distilled water) as previously described [20]. Diameters were determined following incubation at 30°C for up to 24 h. The experiments were repeated at least three times.

### Biofilm formation assay

Biofilm formation was determined using polyvinylchloride (PVC) microtitre plate assay as described previously, with slight modifications [46]. Briefly, bacterial strains were grown overnight in LB at 28°C with agitation and diluted (1∶150) in LB broth. Aliquots of 150 µl of the diluted culture were added to the wells of a PVC microtitre plate, and the plate was incubated at 28°C without shaking. After 48 h, the planktonic bacteria were carefully removed by pipetting, and the wells were washed with water. To visualize biofilm formation, 200 µl of 1% (w/v) crystal violet (CV) was added to each well, and the wells were incubated at room temperature for 5 min before rinsing in tap water. To quantify biofilm formation, CV was dissolved into 200 µl of acetone-ethanol (20∶80), and CV concentration was determined by measuring the OD_540_ of a 100 µl sample diluted in one ml of H_2_O. The experiment was repeated at least three times with three replicates.

### Acid resistance assay

To test for acid resistance, cells were grown in LB for 22 h, collected by centrifugation at 4000 rpm for 10 min, and washed with PBS for three times. Pellets were re-suspended in 200 µl PBS, and 10-fold serial dilutions of bacterial suspensions were made into pre-warmed EG medium pH 2.5 to test acid resistance. Viable counts were determined at time 0, 1, 2, 3 and 4 h after acid challenge. The experiment was repeated at least three times.

### RNA isolation

Bacterial strains were grown overnight in LB broth supplemented with or without antibiotics and diluted in 5 mL M9 media at an OD_600_ of 0.005. After eight hours growth at 34°C in M9 medium, 2 mL of RNA Protect Reagent (Qiagen) was added to 1 ml bacterial cultures (at OD_600_ of about 0.5–0.8) to stabilize RNA. Cells were then harvested by centrifugation for 10 min at 4000 *g* and RNA was extracted using Qiagen Bacterial RNA Mini Kit. Dnase (Qiagen, Hilden, Germany) was used to eliminate residue genomic DNA by an on-column digestion method. RNA integrity was evaluated using the Agilent 2100 Bioanalyzer (Agilent Technologies, Palo Alto, CA, USA) according to manufacturer's instructions.

### Quantitative real-time PCR (qRT-PCR)

qRT-PCR was performed to compare the relative expression of target genes of *E. coli ybjN* mutant or *ybjN* over-expression strain with the WT strain. One microgram of total RNA was reverse-transcribed in a 20 µl reaction using SuperScript® III Reverse Transcriptase (Invitrogen, Carlsbad, CA, USA) following the manufacturer's instructions. For each sample, negative reverse transcription reaction was done to verify the absence of genomic contamination in subsequent qPCR. Primer sequences (Table 1) were designed using Primer3 (http://frodo.wi.mit.edu/primer3/). BLAST searches were performed to confirm gene specificity and the absence of multi-locus matching at the primer site. SYBRGreen qPCR reactions were performed using the ABI 7300 System (Applied Biosystems) in 96 well optical reaction plates. One µl of cDNA (2 ng/reaction) or water (no-template control) were used as template for qPCR reactions with Power SYBR Green PCR Master Mix (Applied Biosystems) and primers at 500 nM final concentration. Primer pairs ybjNEc1–ybjNEc2, ybjNEa1–YbjNEa2, 16S1–16S2, fliA1–fliA2, flgD1–flgD2, fimD1–fimD2, gadA1–gadA2, gadB1–gadB2, gadE1–gadE2, msqR1–msqR2, ygiT1–ygiT2, relB1–relB2 and relE1–relE2 were used to detect the expression of *E. coli ybjN*, *Erwinia ybjN*, *E. coli rrsA*, *fliA*, *flgD*, *fimD*, *gadA*, *gadB*, *gadE*, *msqR*, *ygiT*, *relB* and *relE* genes, respectively. qPCR amplifications were carried out (a cycle of 95°C for 10 min, followed by 40 cycles of 95°C for 15 sec and 60°C for 1 min, and a final dissociation curve analysis step from 65°C to 95°C). Technical replicate experiments were performed for each biological triplicate sample. Amplification specificity for each qPCR reaction was confirmed by the dissociation curve analysis. Determined Ct values were then exploited for further analysis.

Gene expression levels were analyzed using the relative quantification (ΔΔCt) method. A 16S rRNA *rrsA* was used as the housekeeping gene to normalize our samples (ΔCt = *Ct*_target_−*Ct*_rssA_). A relative quantification (RQ) value was calculated as 2 exp-(ΔΔCt = ΔC*t*_target_−Δ*Ct*_reference_) for each gene with the control group as a reference. A p-value was computed using a moderated t-test to measure the significance associated with each RQ value. Variations were considered statistically significant when the p-value was <0.05. RQ values for *ybjN* mutant and over-expression strains were then normalized to those of WT.

### Microarray analysis

Total RNA (10 µg) from each of three biological replicate samples were reverse transcribed and labeled by Alexa Fluor 555 using the FairPlay III Microarray Labeling Kit (Stratagene, La Jolla, CA, USA) according to manufacturer's instruction, except that purification steps were done using the QIAquick PCR mini column system (Qiagen, Hilden, Germany). Labeling efficiency and product integrity was checked by using the microarray preset for the Nano-drop ND-1000 (NanoDrop Technologies, Inc., Wilmington, DE, USA). The hybridization experiment was performed using 0.6 µg Alexa Fluor 555-labeled cDNA in the presence of a 2× hybridization buffer (Agilent technologies, Santa Clara, CA, USA) on the Agilent *E. coli* microarray (8×15K slide format, design ID 020097) for 17 h at 65°C, in a rotating oven (10 rpm). Hybridized slides were first washed for 1 min in a Gene Expression Wash Buffer 1 (Agilent technologies, Santa Clara, CA, USA) at room temperature, then for another 1 min in Gene Expression Wash Buffer 2 (Agilent technologies, Santa Clara, CA, USA) at 37°C. Slides were scanned using an Agilent Scanner (Agilent technologies, Santa Clara, CA, USA) at 5-µm resolution. All slides were scanned using 100% laser power; PMT voltages were automatically adjusted using the Genepix Pro 6.0 software acquisition system to obtain maximal signal intensities with <0.02% probe saturation. The resulting 16 bit images were processed using the GenePix Pro 6.0 image analysis software (v6.0.1.26). Raw data were normalized using the glowess method. The experimental design and microarray data are available at NCBI Gene Expression Omnibus (GEO) (http://www.ncbi.nlm.nih.gov/geo, accession # GSE28630).

Statistical comparisons were performed using multiple testing procedures to evaluate statistical significance for differentially expressed genes. A modified *t*-test (*p*-value) was computed to measure the significance associated with each differential expression value. A gene expression value was decided to be significantly different in the mutant and over-expression strains when the *p*-value was less than 0.05 (except otherwise mentioned) and the expression ratio was ≥2.0 or ≤0.5. Gene functions were assigned using data from EcoCyc (http://ecocyc.org/).

## Supporting Information

Figure S1 **Gene context and protein conservation of** ***ybjN*** **in** ***E. coli*** **,** ***E. amylovora*** **and other related enteric bacteria.** Indicate the accession number in GenBank and bacterial strains.
(EPS)

Figure S2 **Amino acid sequence alignments of YbjN homologs from seven enterobacterial strains.** Deduced amino acid sequences have been obtained from NCBI and aligned by ClustalW program (www.ebi.ac.uk/Tools/clustalw2). Identical residues (*), conserved (**:**) and semi-conserved (**.**) substitutions are shown as underneath symbols. Gaps introduced for alignment are indicated by dashes (-).
(EPS)

Table S1 **YbjN homologues in Enterobacteriaceae that are pathogens or commensals.**
(DOC)

Table S2 **Differentially expressed genes in** ***ybjN*** **mutant and over-expression strains compared to** ***E. coli*** **K-12 strain BW25113.**
(XLSX)

Table S3 **Amino acid and nucleotide biosynthetic genes that are suppressed in** ***ybjN*** **over-expression strain (p-value<0.05).**
(DOC)

Table S4 **Bacterial strains, plasmids and primers used in this study.**
(DOC)
