# Supplementary material for: The Orphan Gene ybjN Conveys Pleiotropic Effects on Multicellular Behavior and Survival of Escherichia coli
Source: PLoS One. 2011 Sep 27;6(9):e25293. doi: 10.1371/journal.pone.0025293 (PMC3181261; doi:10.1371/journal.pone.0025293)
Supplement: Table S1 — YbjN homologues in Enterobacteriaceae that are pathogens or commensals. (DOC) [file pone.0025293.s003.doc]

**Table S1. YbjN homologues in Enterobacteriaceae that are pathogens or commensals**

| **Enterobacteriaceae** | **Host** | **Accession number** | **Identity to YbjN** |
| --- | --- | --- | --- |
| Escherichia coli *str. O157:H7* | Mammals | GI:13360392 | 158/158 (100%) |
| Shigella dysenteriae *2002017* | Mammals | GI:281600176 | 157/158 (99%) |
| Salmonella enterica *serovar Typhimurium str. LT2* | Mammals | GI:16419384 | 134/158 (84%) |
| Yersinia pestis KIM 10 | Fleas and Mammals | GI:21959749 | 108/157 (68%) |
| *Erwinia pyrifoliae Ep1/96* | Pears | GI:259908955 | 108/159 (67%) |
| *Erwinia amylovora ATCC 49946* | Rosacous plants | GI:292899017 | 106/159 (66%) |
| *Pectobacterium atrosepticum SCRI1043* | Potato | GI:50121602 | 104/146 (71%) |
| *Dickeya dadantii 3937* | Pea Aphid  and crops | GI:307130756 | 96/146 (65%) |
| *Photorhabdus luminescens subsp. laumondii TTO1* | Insect larvae | GI:37525530 | 95/140 (67%) |
| *Proteus mirabilis HI4320* | Humans | GI:197284572 | 70/133 (52%) |
