# Supplementary material for: The Orphan Gene ybjN Conveys Pleiotropic Effects on Multicellular Behavior and Survival of Escherichia coli
Source: PLoS One. 2011 Sep 27;6(9):e25293. doi: 10.1371/journal.pone.0025293 (PMC3181261; doi:10.1371/journal.pone.0025293)
Supplement: Table S3 — Amino acid and nucleotide biosynthetic genes that are suppressed in ybjN over-expression strain (p-value<0.05). (DOC) [file pone.0025293.s005.doc]

**Table S3. Amino acid and nucleotide biosynthetic genes that are suppressed in *ybjN* over-expression strain (p-value < 0.05).**

| **Gene Name** | **Gene Function** | ***ybjN*_over-expression/BW** |
| --- | --- | --- |
| Biotin biosynthesis | | |
| *bioA* | 7,8-diaminopelargonic acid synthase | 0.22 |
| *bioB* | biotin synthase | 0.13 |
| *bioC* | predicted methltransferase, enzyme of biotin synthesis | 0.17 |
| *bioD* | dethiobiotin synthetase | 0.20 |
| *bioF* | 8-amino-7-oxononanoate synthase | 0.08 |
| Aspartate and glutamate metabolism | | |
| *asnA* | Asparagine synthase | 0.38 |
| *asnC* | Regulator for asnAC | 0.46 |
| *glnA* | glutamine synthetase | 0.27 |
| *glnG* | Response regulator for gln | 0.32 |
| *glnL* | Histidine kinase for GlnG | 0.30 |
| *ybaS* | glutaminase | 0.22 |
| *gdhA* | glutamate dehydrogenase, NADP-specific | 0.14 |
| *gltD* | glutamate synthase, 4Fe-4S protein, small subunit | 0.33 |
| *gltF* | periplasmic protein | 0.32 |
| Histidine biosynthesis | | |
| *hisC* | histidinol-phosphate aminotransferase | 0.44 |
| *hisD* | bifunctional histidinal dehydrogenase | 0.46 |
| *hisG* | ATP phosphoribosyltransferase | 0.44 |
| *hisI* | fused phosphoribosyl-AMP cyclohydrolase/phosphoribosyl-ATP pyrophosphatase | 0.42 |
| Valine and isoleusine biosynthesis | | |
| *ilvB* | acetolactate synthase III, large subunit | 0.07 |
| *ilvC* | ketol-acid reductoisomerase, NAD(P)-binding | 0.06 |
| *ilvH* | acetolactate synthase III, thiamin-dependent, small subunit | 0.21 |
| *ilvI* | acetolactate synthase III, large subunit | 0.24 |
| *ilvN* | acetolactate synthase III, small subunit | 0.06 |
| Methionine Biosynthesis | | |
| *metA* | homoserine O-transsuccinylase | 0.22 |
| *metC* | cystathionine beta-lyase, PLP-dependent | 0.37 |
| *metE* | 5-methyltetrahydropteroyltriglutamate-homocysteine S-methyltransferase | 0.23 |
| *metH* | homocysteine-N5-methyltetrahydrofolate transmethylase | 0.28 |
| Tryptophen Biosynthesis | | |
| *trpA* | tryptophan synthase, alpha subunit | 0.46 |
| *trpB* | tryptophan synthase, beta subunit | 0.51 |
| *trpC* | fused indole-3-glycerolphosphate synthetase/N-(5-phosphoribosyl)anthranilate isomerase | 0.43 |
| *trpD* | fused glutamine amidotransferase (component II) of anthranilate synthase/anthranilate phosphoribosyl transferase | 0.07 |
| *trpE* | component I of anthranilate synthase | 0.06 |
| Nuleotide biosynthesis | | |
| *guaA* | GMP synthetase (glutamine aminotransferase) | 0.34 |
| *guaB* | IMP dehydrogenase | 0.28 |
| *pntA* | pyridine nucleotide transhydrogenase, alpha subunit | 0.17 |
| *pntB* | pyridine nucleotide transhydrogenase, beta subunit | 0.20 |
| Purine biosynthesis | | |
| *purB* | adenylosuccinate lyase | 0.40 |
| *purC* | phosphoribosylaminoimidazole-succinocarboxamide synthetase | 0.33 |
| *purD* | phosphoribosylglycinamide synthetase phosphoribosylamine-glycine ligase | 0.17 |
| *purE* | N5-carboxyaminoimidazole ribonucleotide mutase | 0.27 |
| *purF* | amidophosphoribosyltransferase | 0.16 |
| *purH* | fused IMP cyclohydrolase/ phosphoribosyl aminoimidazolecarboxamide formyltransferase | 0.17 |
| *purK* | N5-carboxyaminoimidazole ribonucleotide synthase | 0.27 |
| *purL* | phosphoribosylformyl-glycineamide synthetase | 0.27 |
| *purM* | phosphoribosylaminoimidazole synthetase | 0.17 |
| *purN* | phosphoribosylglycinamide formyltransferase 1 | 0.20 |
| *purT* | phosphoribosylglycinamide formyltransferase 2 | 0.12 |
| P[yrimidine biosynthesis](http://biocyc.org/ECOLI/NEW-IMAGE?object=GO:0006207) | | |
| *pyrB* | aspartate carbamoyltransferase, catalytic subunit | 0.01 |
| *pyrC* | dihydro-orotase | 0.12 |
| *pyrD* | dihydro-orotate oxidase, FMN-linked | 0.12 |
| *pyrF* | orotidine-5'-phosphate decarboxylase | 0.42 |
| Transporter | | |
| *livF* | leucine/isoleucine/valine transporter subunit | 0.05 |
| *livG* | leucine/isoleucine/valine transporter subunit | 0.04 |
| *livH* | leucine/isoleucine/valine transporter subunit | 0.04 |
| *livJ* | leucine/isoleucine/valine transporter subunit | 0.05 |
| *livK* | leucine transporter subunit | 0.05 |
| *livM* | leucine/isoleucine/valine transporter subunit | 0.04 |
