# Supplementary material for: The Orphan Gene ybjN Conveys Pleiotropic Effects on Multicellular Behavior and Survival of Escherichia coli
Source: PLoS One. 2011 Sep 27;6(9):e25293. doi: 10.1371/journal.pone.0025293 (PMC3181261; doi:10.1371/journal.pone.0025293)
Supplement: Table S4 — Bacterial strains, plasmids and primers used in this study. (DOC) [file pone.0025293.s006.doc]

**Table S4**. Bacterial strains, plasmids and primers used in this study

| **Strains, plasmids or primers** | **Relevant characters or sequences (5’—3’)^a^** | **Reference or source** |
| --- | --- | --- |
| *E. coli*  BW25113 | K-12 Derivative, ∆*(araD-araB)567*, ∆*lacZ4787*(::rrnB-3), *λ^-^*, *rph-1*, ∆*(rhaD-rhaB)568*, *hsdR514* | [47] |
| *ΔybjN* | F-, *Δ(araD-araB)567*, *ΔlacZ4787*(::rrnB-3), *λ^-^*, *ΔybjN754::kan*, *rph-1*, *Δ(rhaD-rhaB)568*, *hsdR514* | [47] |
| AJW678 | K-12 Derivative, hi-1 thr-1(Am) leuB6 metF159(Am) rpsL136ΔlacX74 | [48] |
| AJW1939 | AJW678 ackA::Km | [48] |
| AJW2528 | AJW678 ackA::Km rcsC::Tet | [20] |
| ts9 | *leuB6 fhuA2 lacY1 tsx-1 glnV44(AS) gal-6 LAM- hisG1(Fs) argG6 rpsL9 malT1(LamR) xylA7 mtlA2 metB1 coaA1 ilu-1* | [49] |
| DH10B | F^-^ *mcr*A ∆(*mrr*-*hsd*RMS-*mcr*BC) Φ80*lac*Z∆M15 ∆*lac*X74 *rec*A1 *end*A1 *ara*∆139 ∆(*ara*, *leu*)7697 *gal*U *gal*K λ - *rps*L (Str^R^) *nup*G | Invitrogen |
| *Erwinia amylovora* | | |
| Ea1189 | Wild type, isolated from apple | [50] |
| Plasmids | | |
| pGEM ® T-easy | Ap^R^, PCR cloning vector | Promega |
| pYbjN1 | 0.9-kb PCR fragment containing *E. coli* *ybjN* gene in pGEM T-easy vector | This study |
| pYbjN2 | 1.1-kb PCR fragment containing *Erwinia ybjN* gene in pGEM T-easy vector | This study |
| Primers^b^ | | |
| ybjN1 | GGGGCTGGAAGGAATAGAAA | |
| ybjN2 | AACTTTAGCCAGGGTTTGAGA | |
| ybjN3 | CCGGAATTCGTTAGTGCATGAAAACTGTTACCG(EcoRI) | |
| ybjN4 | CGCGGATCCATAGCCCCAGTCATTCATGC (BamHI) | |
| 16S1 | TGTAGCGGTGAAATGCGTAG | |
| 16S2 | CCTCCAAGTCGACATCGTTT | |
| ybjNEc1 | CTGCCCCATATGCAGAATTT | |
| ybjNEc2 | TCAGCGAACTGGCATTGAT | |
| ybjNEa1 | TAATGGACGGGGTTATCCTG | |
| ybjNEa2 | ATCAGCTTGGGCAGATTGTC | |
| fliA1 | ACAAGGAACGGCATTTACAA | |
| fliA2 | CCAAGTTCCTGCTCCAGTTG | |
| flgD1 | ACAGGCCAGTAACCTGATCG | |
| flgD2  fimD1  fimD2 | GTGATGGTGGCCGTAACTTT  GTTGATGCAGGCTCTGTTGA  AGGCAACAGCGGCTTTAGAT | |
| gadA1 | CGGATAAACCAAACCTGGTG | |
| gadA2 | TGTTTTCGTCACAGGCTTCA | |
| gadB1 | CGGATAAACCAAACCTGGTG | |
| gadB2 | TGTTTTCGTCACAGGCTTCA | |
| gadE1 | GCCGCAAAGAAAGTATCAAAA | |
| gadE2 | AGCGTCGACGTGATATTGCT | |
| mqsR1 | ACGCACACCACATACACGTT | |
| mqsR2 | CCTGTAACAAGCCTGGGTCT | |
| ygiT1 | ATGCTTTCATGGCGCAAGTA | |
| ygiT2 | GCGAAAACGCATTTACACCT | |
| relB1 | GCGCTTCGTCTCATGCTC | |
| relB2 | AGAGTTCATCCAGCGTCACA | |
| relE1 | GCAAACAAGCTCCGTGGTAT | |
| relE2 | CCGCCTCGCTATATACTTCC | |

^a^ Km^R^, Ap^R^ and Str^R^ = kanamycin, ampicillin and streptomycin resistance, respectively.

^b^Underlined nucleotides are restriction sites added and the restriction enzymes are indicated at the end of primers.
